# Cytokine production in Ancylostoma *duodenale* infection

**DOI:** 10.25122/jml-2021-0383

**Published:** 2022-04

**Authors:** Shaimaa Abdulhussein Shlash, Zubaida Falih Alzubaidi, Huda Ali Saleh

**Affiliations:** 1. Department of Clinical Laboratory Sciences, Faculty of Pharmacy, University of Kufa, Kufa, Iraq

**Keywords:** Ancylostoma duodenale, cytokines, piperazine treatment

## Abstract

Cytokine response to *Ancylostoma duodenale* (*A. duodenale*) infection was measured after starting treatments with piperazine. This study aims to determine the impact of cytokine production after infection with *A. duodenale* before and after treatment with piperazine. Blood and stool samples of 50 patients with *A. duodenale* infection and 28 healthy individuals (control) were collected. In this study, IFNγ, IL-5, IL-12, and IL-13 in serum (using ELISA-based methods) were measured. Stool samples were examined using the Kato-Katz technique to detect *A. duodenale* parasites. Blood and stool samples were analyzed 14 days after starting piperazine treatment for *A. duodenale* infection. The medium concentration of IFNγ, IL-5, IL-12, and IL-13 in the serum samples with *A. duodenale* infection is higher than that of the control group. IFNγ, IL-5, IL-12, and IL-13 levels were significantly higher in the infected individuals (10.5±7.4 pg/ml, 14.6±5.1 pg/ml, 8.5±3.2 pg/ml and 13.6±7.5 pg/ml respectively) than the control group (4.7±2.4 pg/ml, 7.8±4.06 pg/ml, 6.3±3.4 pg/ml and 3.5±2.7 pg/ml respectively). Also, piperazine treatment can significantly reduce cytokines levels (IFN-γ: P=0.043, IL-5: P=0.02, and IL-12, p=0.001). This study shows that piperazine treatment can reduce cytokines profiles in patients with *A. duodenale* infection.

## Introduction

*Ancylostoma duodenale* (*A. duodenale*) infection caused by intestinal helminthiasis is caused by hookworms. Most cases are concentrated in tropical and subtropical countries (humans are infected with *A. duodenale* species worldwide) [1]. Iron deficiency is the major clinical sign because these helminths feed on the blood. The early stage of *A. duodenale* infection is related to the production of pro-inflammatory cytokines IFN-γ, Th1 cells [2]. In the final stage, Th2 cytokines can stimulate B cells to produce antibodies [3]. *A. duodenale* infection related to Th2 immunity response can be distinguished through the production of IL-5 and IL-13, enhancing IgE and IgG4 antibody response [4]. The dynamics involved in the immune response of *A. duodenale* infection are the complex connections of cytokines and response mediators, but these complex connections are fairly known (regardless of the frequency of these infections and numerous treatments) [5]. 

Piperazines are a broad class of chemical compounds with several uses in industry and medicine and are pharmacologically identical to the normally occurring neurohormone inhibitors. It is available as a well-known medicine used as a treatment option against anthelmintic infections, and it is very useful to control intestinal parasites [6]. In different studies, piperazine compounds, administered in single doses, were able to discharge adult Ascaridia [7]. Piperazine and its salts, as aminobutyric acid-like salt, induce fickle soft paralysis in nematode parasites [8]. Piperazine is the main chemotherapeutic agent to treat lymphatic filariasis, reducing the concentration of parasites in peripheral circulation [9].

In this study, the levels of IFNγ, IL-5, IL-12, and IL-13 cytokines in the blood serum of patients infected with *A. duodenale* were analyzed and compared to the immune response of the control group. Furthermore, we compared cytokine serum levels pre and post piperazine treatment.

## Material and Methods

### Experiment planning

A case-control study was conducted with patients, in Al-Furat Al-Awsat teaching hospital, in Al-Najaf province, from January 2021 to May 2021. Patients were 30 males and 20 females, aged 18-45, diagnosed with *Ancylostoma duodenale* infection (detection depends on the presence of *A. duodenale* eggs in the stool examined using modified McMaster salt flotation methods). The intensities of *A. duodenale* infection were determined through Kato-Katz fecal thick smear technique [10], and they were expressed as eggs per gram EPG of stool. All infected individuals were treated with single-dose piperazine of 75 mg/kg. The response to this drug was estimated in the stool specimens gathered after treatment for 14 days. The control group included 28 healthy people with no autoimmune disease who did not receive any medications.

### Specimen Collection

After obtaining consent, a 5 ml blood sample was withdrawn from each participant via vein puncture. The plasma was separated by centrifugation at 1000 rpm for 15 min using 5% EDTA anticoagulation tubes. The separated plasma samples were then stored at -70°C for the determination of cytokine levels.

### Cytokines level determination

The cytokines level yields for all supernatants were detected (the samples, at 107 cells/ml, were stored for 5 days, at 70 degrees below zero, so the cultures could multiply for examination and detection of cytokines levels). Cytokines examination was done using commercially ready ELISA kits, which have dual cytokines specific monoclonal antibodies, following the manufacturers' recommendations and instructions (Benders medium order; Vienna, Austria).

### Statistical analysis

Data were analyzed, and the mean±standard deviations were calculated. T-test was used to compare the means of cytokine concentrations for patients and control groups. The data analysis was performed using SPSS software, where the rating equation models for data, distinguishing standard errors, to assess any significant differences in the cytokines levels pre and post piperazine treatment, using Rubin's rule [11].

## Results

Mean concentrations of IFNγ, IL-5, IL-12, and IL-13 in the patients infected with *A. duodenale* were higher than in the control group. There were significant differences in the concentrations of IFNγ (P<0.001) and IL-13 (P<0.05) between the 2 groups (Table 1).

**Table 1.** Comparison of cytokines levels (mean±SD) in patients and the control group.

**Groups Cytokine**	**Control (pg/ml)**	**Patients (pg/ml)**	**T-test**	**P-value**
**IFN-γ**	4.7±2.4	10.5±7.4	5.3	0.0
**IL-5**	7.8±4.06	14.6±5.1	1.6	0.05
**IL-12**	6.3±3.4	8.5±3.2	0.2	0.35
**IL-13**	3.5±2.7	13.6±7.5	3.1	0.051

There was a significant decrease in IFN-γ (β=−0.77; 95% CI [−1.45, −0.05]; P=0.043), IL-5 (β=−0.89; 95% CI [−1.59, −0.21]; P=0.02) and IL-12 (β=−2.86; 95% CI [−3.57, −2.23]; P=0.001), after treatment with piperazine and worms were expelled. There was no significant IL-13 level alteration post piperazine treatment (Table 2).

**Table 2.** Effects of piperazine on cytokines levels (mean±SD) in patients with A. duodenale.

**Cytokine**	**Pre-treated**	**Post-treated**	**β**	**95% CI**	**P-value**
**IFN-γ**	0.43±0.18	−0.12±0.31	−0.77	−1.45, −0.05	0.043
**IL-5**	−0.85±0.14	−1.55±0.21	−0.89	−1.59, −0.21	0.02
**IL-12**	1.34±0.15	−0.83±0.31	−2.86	−3.57, −2.23	0.001
**IL-13**	3.77±0.08	3.89±0.09	−0.09	−0.20, 0.06	0.21

*^β^ – Effects of piperazine on cytokines levels (pg/mL) after 14 days of treatment; CI – Change bootstrapped 95% confidence interval in an immune response.

## Discussion

This study shows a significant increase in IFN-γ, IL-5, IL-12, and IL-13 in serum levels after *A. duodenale* infection. In addition, the eggs of this parasite can stimulate a rapid increase in IFNγ levels corresponding to the immune response. The level of IL-5 stimulation occurs 5 days after infection when the parasite is thought to be in the luminal phase [12]. IL-12 is produced by antigen-presenting cells pending the first infection to develop Th1 polarization [13], but automatic infection, in the case of *A. duodenale*, can lead to the continuation of infection and rise of IL-12 level. IFNγ is a representative of the Th1 cytokine implicated in the rescue of the interaction of cellular pathogens, where the cytokines act on the regulation of antibody output, and MHC increases type II and energizes macrophage output oxygen-free radical and IFN-γ [14].

**Figure 1.**
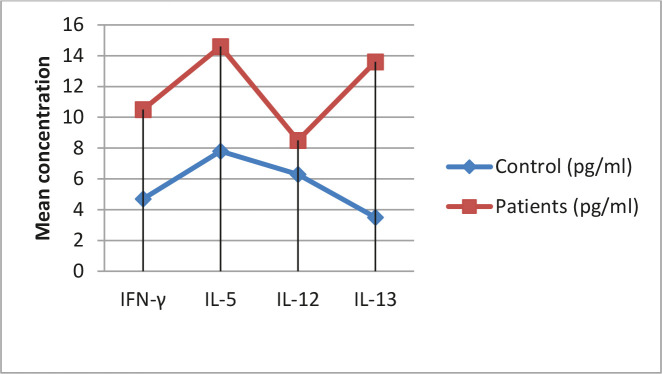
Mean concentration of cytokines levels in patients with A. duodenale and control group.

The anti-inflammatory mediator, such as IL-12, is substantial in saving IFNγ levels to lower the host's potential reverse effect of a long time high IFNγ level [15]. These reactions show why the IL-12 level was high in individuals with *A. duodenale*. These reactions could signal the powerful immunomodulatory effects of *A. duodenale* in patients, thus directing the immune system to an extra immune response appropriate for cytokines' existence [16]. Eotaxin activity is systemic, while IL-5 stimulates a release response of the eosinophils from the bone marrow and intercedes their elected staffing into sore locations (Table 1 and Figure 1) [17].

This study showed a significant reduction in IFN-γ, IL-5, and IL-12 levels in the plasma of patients after treatment with piperazine. Worm expulsion relieves the immune compression and is responsible for increasing cytokine levels, which is caused by returning the cytokines to the rest state. This was not discussed in other studies, like studies that boost the immunity of Th2 cytokine response followed by piperazine treatment in patients with schistosomiasis [18].

**Figure 2.**
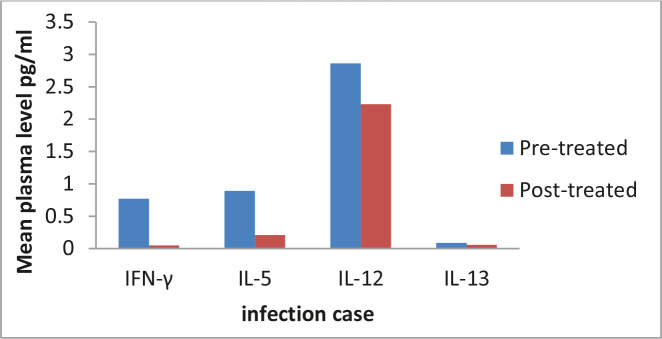
Mean concentration of cytokines levels in patients with A. duodenale and control group.

The mechanism of action of piperazine against the parasite lies in the disturbance of the intravascular and tegument exposition to antigen and metabolic disorder, paralyzing the parasite, and expelling the worms in 14 days. However, there is a significant difference in the mean IL-13 level in the *A. duodenale* group, pre, and post-treatment. The production of enzymes that inactivate exotoxins may be the strategy used by helminths to prevent the induction and effect of eosinophil in the site of infection (Table 2 and Figure 2) [19].

The results of this study are similar to other studies where there were significantly lower IFN-γ responses after treatment due to the immunosuppressive elimination or increased exposition to antigens liberation from dead worms [20]. Also, another study showed the immunosuppressive impact of Th1 because there is no evidence of IL-5 suppression. Furthermore, cumulative IgE is positively correlated with the hookworm load, significantly decreasing post-treatment [21]. Th2 cytokines (IL-13) have been related to low *A. duodenale* load suggesting its protection role [22].

## Conclusion

This study explains that infection with *A. duodenale* can change human cytokines profiles, while post-treatment with piperazine can significantly reduce cytokine levels in patients.

## Acknowledgments

### Conflict of interest

The authors declare no conflict of interest.

### Ethics approval

The study was approved by the Human Research Ethics Committee of the University of Kufa (approval ID: MEC/5.1.2021).

### Consent to participate

Participants received written informed consent before participating in the study.

### Authorship

SAS contributed to data collection. HAS contributed to writing the original draft, methodology, and editing. SAS contributed to conceptualizing the study. ZFA contributed to data analysis and data curation.
